# Probiotic effects on sexual function in women with polycystic ovary syndrome: a double blinded randomized controlled trial

**DOI:** 10.1186/s12905-022-01955-z

**Published:** 2022-09-12

**Authors:** Maryam Azizi-Kutenaee, Solmaz Heidari, Seyed-Abdolvahab Taghavi, Fatemeh Bazarganipour

**Affiliations:** 1 Fertility and Infertility Research Center, Hormozgan University of Medical Sciences, Bandar Abbas, Iran; 2 Medicinal Plants Research Center, Yasuj University of Medical Sciences, Yasuj, Iran

**Keywords:** Probiotic, Letrozole, Sexual function, PCOS

## Abstract

**Background:**

Considering the presence of sexual dysfunction in patients with polycystic ovary syndrome, our aim was to provide scientific evidence studying effect of oral probiotic on sexual function in patients with PCOS treated with letrozole in an Iranian population.

**Methods:**

This study was a double blind randomized clinical trial. Eligible women were allocated to Lactofem plus letrozole (n = 20) or letrozole (n = 20). Folic acid was prescribed to all participants in two groups during the study. In group A, Lactofem capsule was administered daily for the first month. In the second month, letrozole (2.5 mg daily from the third day of the menstrual cycle for 5 days) plus Lactofem capsule same as previous month were prescribed. In group B, letrozole administered 2.5 days from the third day of the menstrual cycle for 5 days in the second month. All questionnaires were completed by patients after the intervention (two months after the start of treatment). The Female Sexual Function Index (FSFI) and Body Image Concern Inventory (BICI) were used to assess sexual function and body satisfaction, respectively.

**Results:**

There is statistically significant increase in chemical pregnancy rate (10% vs. 0%; *p* = 0.05) and clinical pregnancy (10% vs. 0%; *p* = 0.05) in Probiotic plus letrozole group compared to letrozole group after intervention. Based on our results, there was a statistically significant difference in each domain and sum score of sexual function of before and after the intervention between group (*P* < 0.05). Our findings have been shown, after eight weeks, the probiotic plus letrozole group had a significant improvement in the FSFI domains and sum score compared to letrozole alone group. Moreover, there are a significant improvement in mean (IQR) of body image score in probiotic group [30 (4.99) vs. 40 (4.36); *P* < 0.01].

**Conclusion:**

The results of the current study suggests that eight weeks of administration of probiotic may improve chemical and clinical pregnancy rate, sexual function and body satisfaction in women with PCOS.

## Introduction

Polycystic ovary syndrome [1] is the mostly prevalent and complex endocrine disorder that affects women of reproductive age [2]. Research on PCOS has paying attention primarily on improving treatment choices and pregnancy outcomes [1]. Its treatment seems that symptom based and includes insulin sensitizers, contraceptives and progesterone [3, 4]. Letrozole is one of the drugs used to treat PCOS. Letrozole is an active aromatase inhibitor that has good potential for inducing ovulation. Letrozole via the aromatase enzyme inhibits conversion of androgens to estrogen. Thus, the pituitary gland is released from estrogen-negative feedback and FSH is released. It also increases the sensitivity of follicles to this hormone by enhancing the expression of the FSH receptor gene. In addition, it does not adversely affect on the endometrium and cervical mucus, so today it is the first line of treatment in PCOS [5].

Based on recent review, antioxidant and anti-inflammatory therapies could be the future options of treatment for patients with PCOS and it is proposed to consider more drugs in the treatment of PCOS, which should have a profound effect on insulin resistance, improve hyperandrogenemia, infertility, and the overall metabolic environment of women with PCOS [6]. In this regard, recent studies have shown that disturbances in bacterial balance are involved in the pathogenesis of obesity, insulin resistance, and systemic inflammation in various metabolic disorders [7] are considered key factors in the pathogenesis of PCOS. Factors associated with PCOS include insulin resistance and systemic inflammation, which hyperglycemia and pro-inflammatory cytokines also have a synergistic effect on ROS production [8].

Probiotics and synbiotics through metal ion theory, regulation of inflammatory signaling pathways, production of antioxidant metabolites, and reduction of ROS may decrease systemic inflammation. Biomarkers of oxidative stress including MDA, carbonyl protein, TAC, superoxide dismutase (SOD), glutathione peroxidase (GPx) and GSH are increased in women with PCOS. Imbalance in favor of oxidative stress caused by multiple stimuli is strongly associated with the severity of inflammation in PCOS [9]. Increased oxidative events and inflammatory cytokines are associated with an increased risk of hyperandrogenism, insulin resistance, cardiovascular diseases, and diabetes in PCOS [10, 11]. It seems that altering the physiological balance between microorganisms in the intestinal microbiome is involved in the pathophysiology of PCOS, and the use of probiotics may restore this balance. Absorption of probiotics, prebiotics and synbiotics promotes intestinal microbial colony balance and intestinal pH, improves lipid and starch metabolism, improves intestinal digestion and absorbs nutrients by producing inflammatory cytokines. Testosterone and other androgens are significantly increased in women with PCOS. Probably due to excess androgen, which acts as a specific inhibitor of follicle growth in PCOS, increases pre-antral follicle growth but suppresses subsequent follicle growth [12].

Today, in modern centers on women's health due to the serious complications of PCOS, to detection and treatment of this syndrome gets special attention. Current studies show that these patients need more comprehensive treatment; because PCOS can affect the patient's mind, body and identity as a woman [13]. Many of the symptoms of PCOS, such as hirsutism, acne, and amenorrhea, are painful, unpleasant, and unpredictable, with features that are culturally non-feminine and undesirable [14]. Women with PCOS may feel that they are less attractive to their partner and that their partner is less satisfied with their relationship [15]. However, research on this syndrome and the psychological aspects of their sexual function has recently received considerable attention. Research related to sexual function in women with PCOS is limited and often have contradictory results [16]. Sexual function, like PCOS, is a complex biopsychological process. In women with PCOS, sexual function is affected by a variety of factors. What they have found so far is that sexual function is impaired by androgen levels [17], obesity [18], metabolic syndrome and infertility [19], mental health [20], and self-esteem [21]. These factors are common in women with PCOS and can play a role in their sexual dysfunction. Today, foods such as prebiotics and probiotics are widely used because of their health benefits, including reduced insulin resistance, inflammation, and oxidative stress (which increases with PCOS) [22]. Considering the presence of sexual dysfunction in patients with PCOS and the use of letrozole as the first line of treatment in these patients, our aim was to provide scientific evidence as the first RCT studying effect of oral probiotic on sexual function in patients with PCOS treated with letrozole.

## Materials and methods

### Participants

This study was a double blind randomized clinical trial conducted in infertility center of Hormozgan University of Medical Sciences in Bandar Abbas, Hormozgan, Iran.

It was approved by the ethics committee of Hormozgan University of Medical Sciences Ethics Committee, Iran (IR.HUMS.REC.1399.326). After the approval of the ethics committee of Hormozgan medical university of sciences, Iran, this study was conducted in 2020–2021. Participants participated in the study through a notice posted on hospital information boards. Prior to enrollment, informed written consent was obtained from all individuals and all procedures were performed in accordance with the Helsinki Declaration. Inclusion criteria were desire to participate in the study, being 18–38 years of age, married and sexually active, absence of non-classic adrenal hyperplasia, thyroid dysfunction, hyperprolactinemia, non-smoking, having infertility, no problems in speaking or listening, Iranian, not taking any prescription medication (except allergy medications and occasional pain medications) for at least three months before entering the study, having two of the following Rotterdam diagnostic criteria:

1. Polycystic ovaries visualized on ultrasound scan (presence of 12 follicles or more in one or both ovaries and/or increased ovarian volume i.e., > 10 ml),
2. Clinical signs of hyperandrogenism (hirsutism score based on hirsutism score greater than 7 or obvious acne),
3. Having an interval between menstrual periods > 35 days and/or amenorrhea, defined as the absence of vaginal bleeding for at least 6 months (i.e. 199 days).

Subjects were excluded if had unwillingness to participate in the study and previous pregnancy affected by neural tubal defect. This study registered at https://en.irct.ir/user/trial/39789/view with IRCT registration number IRCT20160524028038N8 in 04/10/2020.

### Randomization, hidden distribution, and blindness

Allocation to Lactofem plus letrozole (Group A) or letrozole alone (Group B) was done by “block” randomization method. Randomization tool was statistical software; and hiding method was done by envelopes in the package. Participants and physician were blinded to the allocation. Participants and physician were not aware of group assignment.

### Description of intervention for both groups

It should be noted that the first cycle in patients was dedicated to completed clinical and laboratory evaluation. Moreover; it is necessary to administer folic acid before pregnancy in a person who intends to become pregnant based on Itan ministry of health guideline. Therefore, folic acid (400 μg) was prescribed to all participants in two groups during our research.

- Group A: one Lactofem capsule (including Lactobacillus acidophilus 2 × 10^9^, Bifidobacterium bifidus 2 × 10^9^, Lactobacillus rutri 2 × 10^9^, Lactobacillus fermentum 2 × 10^9^; capsule weight of 500 mg bio-capsule) was administered daily for one month orally. In the second month, letrozole (2.5 mg daily from the third day of the menstrual cycle for 5 days) plus Lactofem capsule same as previous month were prescribed.
- Group B: Letrozole alone administered 2.5 days from the third day of the menstrual cycle for 5 days in the second month.

The patients were evaluated during 12–16 days of menstrual cycles for the response of the drug, the size of the follicles reached in the ovary, and the thickness of the endometrium by vaginal ultrasonography using a 6–9 MHz convex-array transducer (Ultrasonix RP, Vancouver, BC, Canada). When at least two follicles were greater than 18 mm, 250 µg Ovitrelle (MERCK SERONO, Switzerland) intramuscularly was administered for ovulation induction. All questionnaires were completed by patients after the intervention (two months after the start of treatment) under the close supervision of the researcher.

### Outcomes

- Demographic data were collected including:
  - Age
  - Occupation: housewife or employed.
  - BMI: by dividing each patient’s weight by height2 (Kg/M2).
  - Socioeconomic status: The study used years of formal education as a measure of socioeconomic status, categorized into five levels: no education, first level (1 to 5 years), second level (6–9 years), third level (10–12 years) and fourth level (more than 12 years). Studies from Iran showed that education can be a good proxy measure for socioeconomic status for Iranians [23].

- Biochemical pregnancy was defined as a small, transient increase in β-hCG levels. Clinical pregnancies were identified by ultrasound at 5 weeks after HCG injection.
- Number dominant follicles (18 mm) and endometrial thickness were examined by one the first researcher (MA.K).
- *Menstrual history* women were asked about the interval of two menstrual cycles in the last 12 months; their menstrual cycles were classified as following: 21–34-34–60, > 3 months and irregular.
- *Hirsutism* hirsutism scoring was based on the Gallway scale (1961). Hutch et al. [24] modified this scoring system and limited it to 9 androgen sensitive areas each area based on the growth of terminal hair scored from 0 to 4. A score of 7 or more indicated hirsutism (25).
- *Acne* Global Acne Grading Scale (GAGS) was assessed to measure acne. This scale considers six areas of the face, chest and upper back to measure the level of involvement, distribution, density and pilosebaceous units. Each of the six areas scores from 0–4 with the most severe lesion in each area determining the score of that area; the score of each region is multiplied by the factor score. The factor score is calculated according to the area involved: forehead: 2; left and right cheek: 2; nose: 1;chin: 1; chest and upper back: 3. The total score is obtained by multiplying the factor score by total score of involved area [26].
- *Sexual function* taken as impairment of sexual desire and arousal, dyspareunia and orgasmic dysfunction. The questionnaire of Female sexual function index (FSFI) was used in the present study to evaluate sexual function. FSFI was developed by Rosen et al. to evaluate sexual function over a 4-week period. The 19 items questionnaire measures female sexual function in six independent dimensions indulging sexual desire (four items), arousal (four items), lubrication (four items), orgasm (three items), satisfaction (three items) and pain (three items). The answers are rated from zero to five where a higher score indicates more satisfactory sexual function [27]. The reliability and validity of the questionnaire are approved in Iran (28). According to Iranian version, we considered cut-off of a 26 FSFI score or less to indicate sexual dysfunction.
- *Body image* We used the Body Image Concern Inventory (BICI) to examine body image in our study. It contains 19 items related to dissatisfaction and concern about appearance, reassurance seeking, social concerns and avoidance related to appearance. For each item, respondents were asked to rate how often they had the described feeling or performed the described behavior on a Likert scale anchored by 1 = ‘‘never’’ and 5 = ‘‘always’’. The total score on the scale range from 19 to 95. The higher scores indicate higher dissatisfaction of body [29]. Psychometric properties of the BICI in Iranian population have been verified [30].
- *Biochemical assessment* Fasting blood samples (15 ml) were collected at baseline and the end of the intervention at Hormozgan university reference laboratory. Serum fasting plasma insulin (μIU/ml), Fasting blood glucose (mg/dl), total testosterone (ng/ml), and LH/FSH concentrations were measured using commercial validated kits (DiaMetra, Milano, Italy) using ELISA (Monobind, USA).

### Sample size

The following formula to calculate the sample size was used following Esmaeilinezhad et al. [31]At least 20 people were estimated for each group (α = 0.05; β = 0.80; µ1: 91.47;µ2; 96.85; S1: 5.69; S2: 5.03).

$$n = \frac{{\left( {z_{1} - \frac{\alpha }{2} + z_{1} - \beta } \right)\left( {s_{1}^{2} + s_{2}^{2} } \right)}}{{\mu_{1} - \mu_{2} }}$$

### Data analysis

Data analysis was done using descriptive statistics (frequency, percent, median, IQR). Mann–Whitney test and x^2^ was used. We used descriptive statistics as well as the Kolmogorov–Smirnov test to analyze the distribution of data. We used non parametric statistical test due to non-normal distribution of data. The statistical program for Social Sciences (SPSS, version 21; SPSS, Chicago, IL). P values were set as 0.05 for all analyses. This manuscript is in accordance with CONSORT guidelines for reporting randomized trials.

## Results

### Sample characteristics

**Fig. 1:**
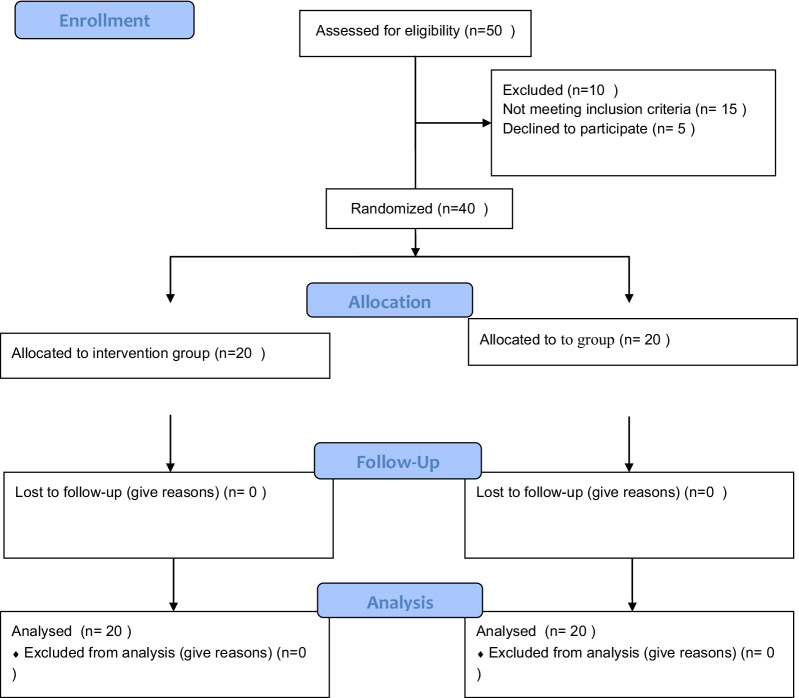
Study flow diagram

Allocation process of participants during the 2020–2021 is shown in Fig. 1. Results of Table 1 show that there is no statistically significant difference between the two groups in age, education, occupation and BMI.

**Table 1:** Socio- demographic of participants

Variable*	Probiotic plus letrozole group (n = 20)	Letrozole group (n = 20)	*P* value
Age of patients*	27.50 (7.25)	27.50 (6.75)	0.91
Education*	12 (4)	12 (1.75)	0.87
BMI*	25.92 (6.46)	24.69 (2.99)	0.07
Occupation**			
Housewife	4 (20)	1 (5)	0.07
Employment	16 (80)	19 (95)	

*Median (IQR)

**n (%)

### Comparison of clinical characterize of PCOS and endocrine laboratory test between group

According to the results of Mann–Whitney test, we did not observe any statistically significant difference in clinical characterize (menstrual interval, hirsutism and acne scores, waist and hip circumstances and weight) and endocrine laboratory tests (LH/FSH ratio, testosterone, insulin and FBS) before and after of intervention of participants between groups (*P* > 0.05). (Table 2 and 3).

**Table 2:** Clinical characterize before and after of intervention

Variable	Probiotic plus letrozole group (n = 20)	Letrozole group (n = 20)	*P* value
Menstrual interval**			
Before			
21–34 day	6 (30)	6 (30)	0.68
35–60 day	7 (35)	5 (25)	
> 3 month	3 (15)	6 (30)	
irregular	4 (20)	3 (15)	
After			
21–34 day	10 (50)	8 (40)	0.16
35–60 day	4 (20)	9 (45)	
> 3 month	3 (15)	0 (0)	
irregular	3 (15)	3 (15)	
Hirsutism score*			
Before	6.5 (1.75)	5.9 (1.32)	0.91
After	5.5 (1.5)	5.4 (1.32)	0.83
Acne score*			
Before	1 (1.2)	1 (1)	0.40
After	0 (0.25)	1 (1)	0.23
Waist circumstance*			
Before	84 (17.5)	84 (13.5)	0.46
After	83.50 (16.87)	83.30 (14.5)	0.41
Hip circumstance*			
Before	100 (13.75)	103 (8.75)	0.64
After	100 (12.75)	103 (8.5)	0.86
Weight *			
Before	74 (15.75)	65.50 (14.5)	0.14
After	73.50 (15.75)	67.75 (14.83)	0.09

*Median (IQR)

**n (%)

**Table 3:** Endocrine laboratory test before and after intervention of participants

Variable*	Probiotic plus letrozole group (n = 20)	Letrozole group (n = 20)	*P* value
*LH/FSH*			
Before	1.87 (0.73)	1.25 (0.94)	0.12
After	1.72 (0.78)	0.80 (0.76)	0.05
*Testosterone*			
Before	1.15 (015)	0.95 (0.73)	0.13
After	0.65 (0.32)	0.93 (0.41)	0.90
*Insulin*			
Before	15.30 (5.32)	10.80 (4.91)	0.81
After	14.30 (5.10)	10.64 (4.83)	0.47
*FBS*			
Before	89 (14.1)	94 (13.85)	0.12
After	89 (13.84)	93.9 (12.99)	0.69

*Median (IQR)

### Comparison of fertility outcomes between groups

We did not observe any statistically significant difference in endometrial thickness before and after intervention and follicle numbers before and after of intervention of participants between groups (*P* > 0.05). (Table 4).

**Table 4:** Fertility outcome of participants

Variable*	Probiotic plus letrozole group (n = 20)	Letrozole group (n = 20)	*P* value
Endometrial thickness *			
Before	7 (1)	7 (0.88)	0.13
After	7 (1)	7 (0.95)	0.82
Follicle numbers*	2 (1)	2 (2)	0.51
Chemical pregnancy**	2 (10)	0	0.05
Clinical pregnancy**	2 (10)	0	0.05

*Median (IQR)

**n (%)

There is statistically significant increase in chemical pregnancy rate (10% vs. 0%; *p* = 0.05) and clinical pregnancy (10% vs. 0%; *p* = 0.05) in probiotic plus letrozole group compared to letrozole group after intervention. (Table 4).

### Comparison of sum score of sexual function and body satisfaction between group

Based on the results of Mann–Whitney test, there was a statistically significant difference in each domain and sum score of sexual function of before and after the intervention between group (*P* < 0.05). Our findings have been shown, after eight weeks, the probiotic plus letrozole group had a significant improvement in the FSFI domains and sum score compared to letrozole alone group.

Moreover, there are a significant improvement in mean (IQR) of body image score in probiotic plus letrozole group [30 (4.99) vs. 40 (4.36); *P* < 0.01]. (Table 5).

**Table 5:** Comparison of sexual function and body image scores before and after of intervention between participates

Variable*	Probiotic plus letrozole group (n = 20)	Letrozole group (n = 20)	*P* value
Domains of FSFI			
Desire			
Before	4.65 (0.31)	4.20 (0.34)	0.09
After	4.80 (0.44)	4.20 (0.61)	0.05
Arousal			
Before	4.75 (0.27)	4.20 (0.60)	0.08
After	4.95 (0.50)	4.35 (0.60)	0.003
Lubrication			
Before	5.10 (0.81)	5 (0.51)	0.21
After	5.49 (0.57)	5 (0.60)	0.05
Orgasm			
Before	5.20 (0.20)	5 (0.21)	0.12
After	5.60 (0.51)	5.20 (0.70)	0.04
Satisfaction			
Before	5.21 (0.31)	5 (0.52)	0.17
After	5.89 (0.59)	5.23 (0.80)	0.05
Pain			
Before	4 (0.29)	3.81 (0.42)	0.45
After	3.60 (0.41)	3.34 (0.23)	0.05
Sum score			
Before	29.35 (5.91)	28.10 (4.79)	0.11
After	29.75 (8.30)	27.40 (6.51)	0.05
Body image			
Before	44.50 (4.75)	42 (5.32)	0.79
After	30 (4.99)	40 (4.36)	0.01

*Median (IQR)

## Discussion

### Effect on infertility

PCOS is associated with chronic ovulation and infertility associated with hormonal / metabolic imbalances including insulin resistance, hyperandrogenism, hypercholesterolemia, and systemic inflammation [32, 33]. Recently, the intestinal microbiome has been shown to play a key role in human health and disease [34]. Gut microbes are many benefits to the host including protection against pathogens and host immune regulation and the integrity of the intestinal barrier integrity [35]. Intestinal microbiome regulates host metabolism, and several intestinal microbiome phenotypes are associated with chronic disease [36–38]. Because the intestinal microbiome regulates various physiological functions important in PCOS (including energy homeostasis, glucose metabolism, systemic inflammation), the intestinal microbiome may be involved in the pathogenesis of PCOS. In addition to human studies, some studies in rodent models have reported a significant relationship between intestinal microbiome and PCOS [39, 40]. In our study, after 8 weeks, the probiotic supplement with letrozole group had a significant increase in the chemical and clinical pregnancy compared to letrozole alone group. Another positive effect of Lactobacillus supplements to reduce inflammation in patients with PCOS and thus improve their fertility reported. This recent study to evaluate the effect of probiotic supplementation in improving inflammation associated with PCOS is done. The results showed a decrease in IL-6 and hs-CRP and a significant increase in IL-10 levels in the experimental group receiving the probiotic supplement compared to the placebo group. On the other hand, no significant change was observed in the levels of TNF-α in the experimental group. Previous results support the role of probiotics in reducing inflammation and thus improving fertility in patients with PCOS [41].

In accordance with the "Dysbiosis of Gut Microbiota" theory, the gut microbiome can activate the host's immune system, causing chronic inflammation that disrupts the function of insulin receptors and leads to insulin resistance. The resulting hyperinsulinemia interferes with follicle formation, while stimulating the production of androgens by ovarian excretory cells [4]. A previous meta-analysis of probiotic/synbiotic PCOS patients showed that probiotic or synbiotic use was associated with decreased fasting plasma glucose ([FPG] mg/dl), fasting blood insulin ([FBI] µIU/mL), and HOMA I-R and a slight improvement in QUICK-I [3].

Our finding is similar to this study. Serum insulin and FBS level were slight improvement in probiotic supplement with letrozole compared to letrozole alone group. But this differences not significant. It’s may be to small sample size in our study. Future studies in with increase sample size will are recommended. Incorrect regulation of glucose metabolism can be the cause of PCOS and may have long term consequence of PCOS. Restoration of the gut microbiome on glucose homeostasis using probiotic and synbiotic show potential impact in improving the absorption of micronutrients in patients with PCOS. Probiotics appear to improve HOMA-IR after 12 weeks of treatment in women with type 2 diabetes [7].

Also, in our study probiotic supplement affect on reduction in the LH/FSH level. This was similar to Heli et al. (2020) that 52 men with idiopathic oligoasthenoteratozoospermia in randomized, double-blind controlled clinical trial randomly assigned to either an intervention or placebo (n = 26) group. The Intervention group took 500 mg of probiotics daily and the placebo group took a daily placebo for 10 weeks. Probiotic supplement decreased LH level but this difference was not significant (5.89 ± 1.75 vs. 5.03 ± 1.14; *P* = 0.10) [42].

### Effect on sexual function

Our findings have been shown, after eight weeks, the probiotic supplement with letrozole had a significant improvement in the FSFI domains and body image compared to letrozole alone group. Body image is the mental picture of a person's body, attitude about physical appearance and health, integrity, normal functioning and gender is defined. Body image is a component of a larger concept that includes a sense of femininity and attractiveness to women [43–47], a symbol of social expression, and as a way of being in the world [46]. Human sexuality involves sexual activity, an ever-changing experience that affects how one looks at oneself, the body, and sexuality [48]. Scientists have shown that the disease causes the body to lose control and feel useless and leads to a negative perception of the body [49]. Infertility is a physical disability and women feel that they are suffering from a physical disability. Therefore, the question arises that infertility (similar to chronic diseases) can be associated with changes in body image [50]. Recently reported that infertile women with PCOS have body dissatisfaction than women with infertility due to other factors that support the contribution of other factors other than infertility associated with PCOS [51].

In the other hand, in our study, probiotic supplement with letrozole group, LH/FSH ratio significantly decreased compared to letrozole alone group. In previous studies, LH was shown to be connected with orgasm problems in healthy and pain, postmenopausal women [32]. More studies are needed to provide supports for the results observed in the current study.

It seems that due to the importance of infertility in our country, with the success of fertility, body image and sexual function in the probiotic supplement with letrozole significantly improved compared to the letrozole group. It should be noted that the burden of infertility in patients varies based on socio-cultural factors, traditions and religious beliefs. Our study was conducted in a developing country where common people are Muslims. Unlike Western culture, in our culture (especially in traditional beliefs) marriage is based on family arrangements. Therefore, considering the most Islamic countries have a male-centered family system, low marital satisfaction is a frequent problem, particularly among women. Based on Islamic beliefs, marriage is the most important way to reproduce and ensure the formation of a family [52]. Therefore, Iranian infertile women are under a lot of force to reproduce and infertility may affect them more than women from Western cultures [53–55]. The findings of the present study are generally consistent with previous studies by Winkwist and Akhtar [56]. Many Pakistani Islamic women feel that their pattern of childbearing influences how people treat them. When they have children in the family are respected. Without children, they do not feel like real women [56, 57]. Women with different ethnic and cultural backgrounds are likely to have different responses and perceptions of their bodies and sexual function, and this provides a potential reason for this difference in the reported problem. For example, Schmid and colleagues (2004) reported that Muslim women reported their menstruation and infertility as the most important concern, while non-Muslim European women had a completely different idea. Obesity with a male pattern was the most important problem in the mentioned patients. This may be explained due to the idea of unattractiveness and clothing models in western culture; While, in the eastern culture, it is considered as a symbol of prosperity to some extent [58]. Also, according to Islamic rules, women must cover their bodies. Based on this, the majority of young Iranian women try to keep their bodies away from others by wearing long and loose clothes in public places (compared to the common clothing worn by women in Western countries). This can lead to a lower level of stress about body shape and more satisfaction from it; this matter point in the research of Rastmenesh and colleagues [59], which shows that those Iranian women who used more complete hijab and covering, reported more self-esteem and body satisfaction [59]. This issue of how to cover up in public may have an effect on the extent of the problem of hirsutism in Muslim women compared to others. Therefore, based on previous work, keeping in mind the importance that is placed on physical appearance in women in the majority of cultures, it is easy to understand why it is that women have the most problems when it comes to body image and more research in this field is needed in relation to cross-cultural comparison in this population.

There are some limitations. First, because the data were reported by the participants themselves, the possibility of incorrect reporting could not be completely ruled out. In addition, patients were participated from a gynecological clinic and it was found that the population of patients undergoing medical treatment or under medical treatment differed in terms of the number of socio-cultural and psychological parameters compared to the population sample. Moreover, it can be argued that there is a bias towards PCOS patients with menstrual disorders and infertility, rather than those with other PCOS-related symptoms (e.g., weight gain and acne) that often go to endocrine clinics. However, it was felt that enough patients with skin or endocrine problems would allow us to study. In addition, all patients in this study due to cultural reasons in Iran were married. So, the results of the current study should be interpreted with caution. Also, the small sample size of the interventions performed may indicate additional sources of bias. Currently, further studies with larger samples, including community and other participants in Iran and ethnic groups, are needed. In addition, there is a clear need for a well-guided RCT structure to demonstrate the potential effects related to fertility and sexual function of the probiotic supplement.

### Conclusion

In conclusion, allowing for the limitations mentioned above, the results of the current first RCT study suggests that eight weeks of administration of probiotic may reduce LH/FSH ratio and improve chemical and clinical pregnancy rate, sexual function and body satisfaction in women with PCOS.

## Data Availability

The primary data for this study is available from the corresponding author (FB) on direct request.
